# Perception of the COVID-19 Pandemic Among Members of Saudi Society: Solidarity, Humility, and Connectivity

**DOI:** 10.7759/cureus.19427

**Published:** 2021-11-10

**Authors:** Baraa Alghalyini, Alaa Albeyahi, Bader Abou Shaar, Mohamed Salah

**Affiliations:** 1 Family and Community Medicine, College of Medicine, Alfaisal University, Riyadh, SAU; 2 Emergency Medicine, King Faisal Specialist Hospital and Research Centre, Riyadh, SAU; 3 Family & Community Medicine, University of Toronto, Toronto, CAN; 4 Public Health, Saudi Ministry of Health, Riyadh, SAU; 5 Public Health, College of Medicine, Alfaisal University, Riyadh, SAU

**Keywords:** community connectivity, community perception, solidarity, wearing gloves, masks, sneezing etiquette, handwashing, social distancing, precautionary measures, covid-19

## Abstract

Background and purpose

The magnitude of the coronavirus disease 2019 (COVID-19) pandemic on the healthcare system, economy, education, and social networking is dreadful, the least to say. Surprisingly, and unlike previous epidemics, the impact has been universal, and even top-ranking countries with solid economies were not immune. The purpose of this study is to develop a better understanding of the Saudi community's response and reaction to the preventative measures implemented by the government to combat the COVID-19 pandemic.

Methodology

A cross-sectional study using a self-administered online-based questionnaire was conducted among 920 participants from March 2020 to February 2021 among the Saudi community across the Kingdom.

Results

Among the studied participants, the majority (60%) are always committed to washing their hands according to the Ministry of Health (MoH) instructions, and 74% indicated that they were always compliant with the sneezing etiquette outlined by the MoH. Studied participants were affected through different influencers of life aspects. Moreover, 63% of them gained new skills and behaviors during the pandemic curfew. Additionally, many studied participants assumed that "life will not return to what it used to be" as a future perception.

Conclusion

In conclusion, the present findings proved the importance and power of the Saudi Vision (2030) represented by the National Transformation Program on enhancing the healthcare system, facilitating access to healthcare, and integrating technology among government parties addressed during the COVID-19 pandemic.

## Introduction

The new coronavirus disease 2019 (COVID-19) caused by severe acute respiratory syndrome coronavirus 2 (SARS-CoV-2) is an extremely infectious disease that was declared a pandemic on March 11, 2020. Since then, the world has become a completely different place as COVID-19 brought nations to a standstill, drove hospital systems to the brink, and dragged the global economy to stagnation [1-2]. An explosive rise in the number of cases and associated mortality in addition to the lack of definitive treatment and vaccine options urged many governments and health authorities to implement strict precautionary measures to combat the pandemic. These measures included travel and movement restrictions, community lockdown, and cancelation of events and non-essential gatherings [3]. Such measures may help postpone the exponential spread of the disease until a vaccine becomes available. Trust and transparency are critical to preserving a calm and compliant response to mitigation advice among the public [4]. Data suggest that countries that implemented early and resolute interventions may have assisted in flattening their epidemic curves and reducing the rapid spread of the disease [4-5]. The Saudi government equipped itself with decisive, yet culturally tailored, precautionary measures and undertook various health policies to curb the pandemic before its beginning in the Kingdom on March 2, 2020, when the first case of COVID-19 was documented in Saudi Arabia [6-7]. A study done in April 2020 indicated that Saudi Arabia has taken bold measures to combat COVID-19, but the consequences of these actions are yet to be studied [7]. However, the success of these interventions and the compliance of the public with them is undoubtedly linked to efficient risk communication with the public to convey the reasoning behind the decisive actions.

Soon after, on March 8, the Saudi Ministry of Education declared the closure of schools, universities, and other institutions until further notice, to avoid the spread of the disease through these congregated settings [8]. The decision covered all educational institutions, including public and private schools and technical and vocational training establishments [7]. This caused an abrupt shift and a transition to alternative online teaching methods [9].

While cooperation is a crucial element to combat the COVID-19 pandemic across the world, solidarity is too. There are examples of solidarity among different countries globally, which are gathering the world's leading experts for discussions on top research and innovation and working to promote knowledge and empower communities with state members. These plans were developed by the WHO based on solidarity [10]. The successful COVID-19 pandemic response in Vietnam emphasized the care and solidarity among its population through compliance with precautionary measures under the care ethics [11].

Locally, Saudi Arabia has approached the facilitation of free healthcare access for everyone in its community, especially for the vulnerable migrant population residing illegally [12]. Social media helped spread solidarity in the Saudi population and has kept their response positive towards the pandemic since the beginning. However, this effect began to decline over time. A recent study showed how people supported each other during the pandemic through social media by raising trendy hashtags on the Twitter platform [13]. Furthermore, the Saudi government designated March 2 of every year as "Health Martyr Day" as a sign of solidarity and appreciation to all healthcare workers who have lost their lives on the frontline against COVID-19 [14]. Saudi Arabia has also declared that free COVID-19 vaccines will be provided to all its residents and nationals and will make them available in pharmacies [15].

The purpose of this study was to assess the Saudi community's perception of the precautionary measures and explore the community’s solidarity towards the implementation of these measures during the COVID-19 pandemic.

## Materials and methods

A cross-sectional study was conducted from March 2020 to February 2021 among Saudi individuals across the Kingdom. Study participants were selected, and probability sampling was applied according to the reported population size in the Saudi Arabia national census in 2019, which was 34,218,169 [16]. This sampling technique helps us generalize our sampling in the Kingdom.

OpenEpi software version 3 (www.OpenEpi.com) was used to calculate the sample size with a maximum possible variability at the 50% and 99% confidence interval level [17]. However, all individuals living in Saudi Arabia and individuals 15 years old and above were included. Nevertheless, all Saudi and non-Saudi individuals not currently living in Saudi Arabia and individuals below 15 years of age were excluded.

The self-administered online-based questionnaire was disseminated to Saudi community individuals through social media in May 2020 before the curfew unblock. As the study tool was generated by the study researcher, a pilot test was done on 20 participants, 10 persons for each language, before disseminating the questionnaire to the public. Lately, Saudi society has become more accustomed to technology and the internet, yet a low response rate might be anticipated. Low and non-response are due to people's willingness to answer the questionnaire and not a mean sample bias [18].

Participants were invited to enter a prize draw of up to 10 gift vouchers chosen randomly to overcome this anticipated barrier. Prizes were selected based on their phone numbers and email address to ensure any repetition within the participants using the Wheel of Names website [19].

The SurveyMonkey website was used for generating the questionnaire [20]. It comprised four sections, with 23 (open and close-ended) questions: demographic characteristics, questions towards the crisis influence on religion, income, health, and future perception and expectations. These questions included branching logics to minimize the length of a questionnaire and provided in both the Arabic and English languages, as shown in the Appendix.

Informed consent was written as an introduction at the beginning of the questionnaire, explaining the research purpose and ethical considerations. The information taken was kept confidential and will not be used for purposes other than the study. Those who did not meet the inclusion criteria were excluded. This study was approved by Alfaisal University's Institutional Review Board with the number IRB-20035.

Data were cleaned using Microsoft Excel (version 2016; Microsoft Corporation, Redmond, WA) and then analyzed using SPSS (version 26; IBM Corp., Armonk, NY) [21-22]. Estimates were reported using frequencies and proportions while significance was tested using the chi-square test. Statistical significance was determined based on a p-value of <0.05, and results were determined as highly statistically significant for a p-value of less than <0.01. This study's dependent variable is combating COVID-19 preventive measures while the independent variables include demographic characteristics, prevention practices, and behaviors from the COVID-19 pandemic crisis effect.

## Results

This study included 920 participants who fully completed the questionnaire as shown in Table 1.

**Table 1:** The demographic characteristics of the studied participants

Demographic Characteristics		Count	%
Age	15-25 years old	226	24.6%
	26-36 years old	302	32.8%
	37-47 years old	221	24.0%
	48-58 years old	132	14.3%
	59 years old and above	39	4.2%
	Total	920	100.0%
Gender	Male	319	34.7%
	Female	601	65.3%
	Total	920	100.0%
Nationality	Saudi	877	95.3%
	Non-Saudi	43	4.7%
	Total	920	100.0%
Kingdom Region	Central Region	722	78.5%
	Southern Region	64	7.0%
	Eastern Region	49	5.3%
	Northern Region	14	1.5%
	Western Region	71	7.7%
	Total	920	100.0%
Marital Status	Married	512	55.7%
	Unmarried	408	44.3%
	Total	920	100.0%
Monthly Income	Less than 5,000 Saudi Riyals	177	19.2%
	5,000 – 10,000 Saudi Riyals	212	23.0%
	11,000 – 15,000 Saudi Riyals	162	17.6%
	More than 15,000 Saudi Riyals	215	23.4%
	I don't have a monthly income	154	16.7%
	Total	920	100.0%
Education	Pre-university Education	154	16.7%
	University Education	586	63.7%
	Post-graduate Education	180	19.6%
	Total	920	100.0%
Employment Sector	Governmental	402	437%
	Military	30	3.3%
	Private	171	18.6%
	Employer	11	1.2%
	Student	151	16.4%
	Unemployed/Housewife	108	11.7%
	Retired	22	2.4%
	Others	25	2.7%
	Total	920	100.0%
If you are working in business administration, do you have your employees to lead?	Yes	82	8.9%
	No	838	91.1%
	Total	920	100.0%

The majority (60%) are always committed to washing their hands for 40 seconds and sanitizing them for 20 seconds according to the Ministry of Health (MoH) guidelines. Moreover, 74% of the total population reported being consistently compliant with the proper sneezing etiquette outlined by the MoH; 20% of them usually complied while 5% of them "did not know" about the proper sneezing etiquette.

Furthermore, when comparing gender with the participants' commitment to washing their hands according to the MoH instructions, males reported a higher percentage than females (66% and 58%, respectively) and was statistically significant (p-value .03). When going outside the home, 75% of females committed to using gloves compared to only 61% of males and was also statistically significant (p-value <.001). At the same time, 71% of males used face masks compared to 62% of females, which was highly statically significant (p-value .005). Only 49% of males washed their hands at the workplace compared to 41% of females, which was statically significant (p-value .012). Further, only 6% of females indicated they never leave home during the pandemic, which was statically significant (p-value <.001).

**Table 2:** Participants' perception of the protective measures according to educational levels

Community Perception on protective measures			Education															Chi-square test	
			Pre-university Education				University Education			Post-graduate Education			Total				p-value		
			Count		%		Count		%	Count	%		Count		%				
Did you commit to wash your hands for 40 seconds and sanitize them for 20 seconds according to (Ministry of Health) instructions on “when to wash your hands?”?	Always	85		55%		348		59%		123	68%	556		60.4%		.158b			
	Usually,	36		23%		147		25%		36	20%	219		23.8%					
	I commit to wash my hands but not according to instructions	31		20%		86		15%		20	11%	137		14.9%					
	I did not commit it at all	2		1%		5		1%		1	1%	8		0.9%					
	Total	154		100%		586		100%		180	100%	920		100.0%					
When you go to any place outside your home, what do you do? (Choose all that applied)																			
I use gloves	Yes	113		73%		420		72%		115	64%	648		70.4%		.092b			
	NO	41		27%		166		28%		65	36%	272		29.6%					
	Total	154		100%		586		100%		180	100%	920		100.0%					
I use a face mask	Yes	98		64%		372		63%		132	73%	602		65.4%		.046*b			
	NO	56		36%		214		37%		48	27%	318		34.6%					
	Total	154		100%		586		100%		180	100%	920		100.0%					
I use hand sanitizer when touching things	Yes	97		63%		405		69%		143	79%	645		70.1%		.003**b			
	NO	57		37%		181		31%		37	21%	275		29.9%					
	Total	154		100%		586		100%		180	100%	920		100.0%					
Washing hands with soap at the workplace	Yes	63		41%		249		42%		89	49%	401		43.6%		.197b			
	NO	91		59%		337		58%		91	51%	519		56.4%					
	Total	154		100%		586		100%		180	100%	920		100.0%					
I keep a safe distance from other people	Yes	104		68%		443		76%		148	82%	695		75.5%		.008**b			
	NO	50		32%		143		24%		32	18%	225		24.5%					
	Total	154		100%		586		100%		180	100%	920		100.0%					
I don't do anything	Yes	4		3%		10		2%		4	2%	18		2.0%		.746b			
	NO	150		97%		576		98%		176	98%	902		98.0%					
	Total	154		100%		586		100%		180	100%	920		100.0%					
I never leave home	Yes	10		6%		27		5%		2	1%	39		4.2%		.040*b			
	NO	144		94%		559		95%		178	99%	881		95.8%					
	Total	154		100%		586		100%		180	100%	920		100.0%					
Washing hands when soap on returning home	Yes	1		1%		2		0%		1	1%	4		0.4%		.843b			
	NO	153		99%		584		100%		179	99%	916		99.6%					
	Total	154		100%		586		100%		180	100%	920		100.0%					
Take shower on returning home	Yes	0		0%		2		0%		0	0%	2		0.2%		.565b			
	NO	154		100%		584		100%		180	100%	918		99.8%					
	Total	154		100%		586		100%		180	100%	920		100.0%					
I wash & sterilize my hands and clothes	Yes	1		1%		0		0%		0	0%	1		0.1%		.083b			
	NO	153		99%		586		100%		180	100%	919		99.9%					
	Total	154		100%		586		100%		180	100%	920		100.0%					
I sterilize my hands each time I touch something at work	Yes	0		0%		1		0%		0	0%	1		0.1%		.752b			
	NO	154		100%		585		100%		180	100%	919		99.9%					
	Total	154		100%		586		100%		180	100%	920		100.0%					
I don’t touch my face	Yes	0		0%		1		0%		0	0%	1		0.1%		.752b			
	NO	154		100%		585		100%		180	100%	919		99.9%					
	Total	154		100%		586		100%		180	100%	920		100.0%					
I cover my head and feet	Yes	0		0%		1		0%		0	0%	1		0.1%		.752b			
	NO	154		100%		585		100%		180	100%	919		99.9%					
	Total	154		100%		586		100%		180	100%	920		100.0%					
I cover my head only	Yes	0		0%		0		0%		1	1%	1		0.1%		.128b			
	NO	154		100%		586		100%		179	99%	919		99.9%					
	Total	154		100%		586		100%		180	100%	920		100.0%					
I tried to comply	Yes	0		0%		1		0%		1	1%	2		0.2%		.511b			
	NO	154		100%		585		100%		179	99%	918		99.8%					
	Total	154		100%		586		100%		180	100%	920		100.0%					
I use all of them	Yes	0		0%		1		0%		0	0%	1		0.1%		.752b			
	NO	154		100%		585		100%		180	100%	919		99.9%					
	Total	154		100%		586		100%		180	100%	920		100.0%					
Others	Yes	3		2%		6		1%		0	0%	9		1.0%		.193b			
	NO	151		98%		580		99%		180	100%	911		99.0%					
	Total	154		100%		586		100%		180	100%	920		100.0%					
Do you comply with the sneezing etiquette" outlined by the Ministry of Health in cases of sneezing?"	Always	110		71%		430		73%		138	77%	678		73.7%		.460b			
	Usually,	31		20%		116		20%		37	21%	184		20.0%					
	Never	2		1%		9		2%		1	1%	12		1.3%					
	I don’t know it	11		7%		31		5%		4	2%	46		5.0%					
	Total	154		100%		586		100%		180	100%	920		100.0%					
When you receive or bring a thing from outside, what did you do? (Choose all that applied)																			
I use gloves	Yes	86		56%		325		55%		106	59%	517		56.2%		.717b			
	NO	68		44%		261		45%		74	41%	403		43.8%					
	Total	154		100%		586		100%		180	100%	920		100.0%					
I use face mask	Yes	48		31%		156		27%		78	43%	282		30.7%		< .001>			
	NO	106		69%		430		73%		102	57%	638		69.3%					
	Total	154		100%		586		100%		180	100%	920		100.0%					
I keep a safe distance from other people	Yes	81		53%		291		50%		115	64%	487		52.9%		.004**b			
	NO	73		47%		295		50%		65	36%	433		47.1%					
	Total	154		100%		586		100%		180	100%	920		100.0%					
I sterilize my order	Yes	99		64%		426		73%		105	58%	630		68.5%		.001**b			
	NO	55		36%		160		27%		75	42%	290		31.5%					
	Total	154		100%		586		100%		180	100%	920		100.0%					
I get rid of outer cases and cartoons	Yes	120		78%		484		83%		142	79%	746		81.1%		.295b			
	NO	34		22%		102		17%		38	21%	174		18.9%					
	Total	154		100%		586		100%		180	100%	920		100.0%					
I don't do anything	Yes	7		5%		17		3%		6	3%	30		3.3%		.592b			
	NO	147		95%		569		97%		174	97%	890		96.7%					
	Total	154		100%		586		100%		180	100%	920		100.0%					
Others	Yes	7		4.5%		16		2.7%		1	0.6%	24		2.6%					
	NO	147		95.5%		570		97.3%		179	99.4%	896		97.4%					
	Total	154		100%		586		100%		180	100%	920		100.0%					

As presented in Table 2, postgraduates were more likely to always commit to hand-washing compared to pre-university-educated participants (68% to 55%). Furthermore, postgraduates were more likely to indicate always/usually commit to sneezing etiquette compared with pre-university educated participants (77% to 71%). Thus, 59% of postgraduates used gloves, 43% used face masks, and 64% kept a safe distance from other people when going outside the home compared with other educational groups while 73% of the university group were more likely to sterilize their outside home orders compared with other pre-university and postgraduates’ groups (64% to 58%). The associations were highly statically significant for using a face mask, keeping a safe distance, and sterilizing orders.

Moreover, 97% of female participants advised people to follow and commit to the preventive measures. Which was slightly higher compared to males who indicated 94.7%. No significant association was found between gender and advising others to be committed. When researchers asked the participants why they advise others, the most frequent answer was “I care about/love them” for both males and females (82.4%, and 84.4%), respectively. However, more females (18.3%) blamed people around them for not committing and following the preventive measures compared to males (13.2%), and this difference was statistically significant (p-value = 0.046).

Besides, 75.9% of males and 75.2% of females reported calling the Unified MOH Number (937) when they had early symptoms and wanted to self-screen for possible COVID infection via the Mawid application. Upon development of COVID-related symptoms, females were more likely to surf the internet for COVID-19 symptoms, use the self-assessment service via the Mawid application, and go to the nearest health facility compared to males (34% and 21%, respectively) (p-value <0.001).

**Figure 1:**
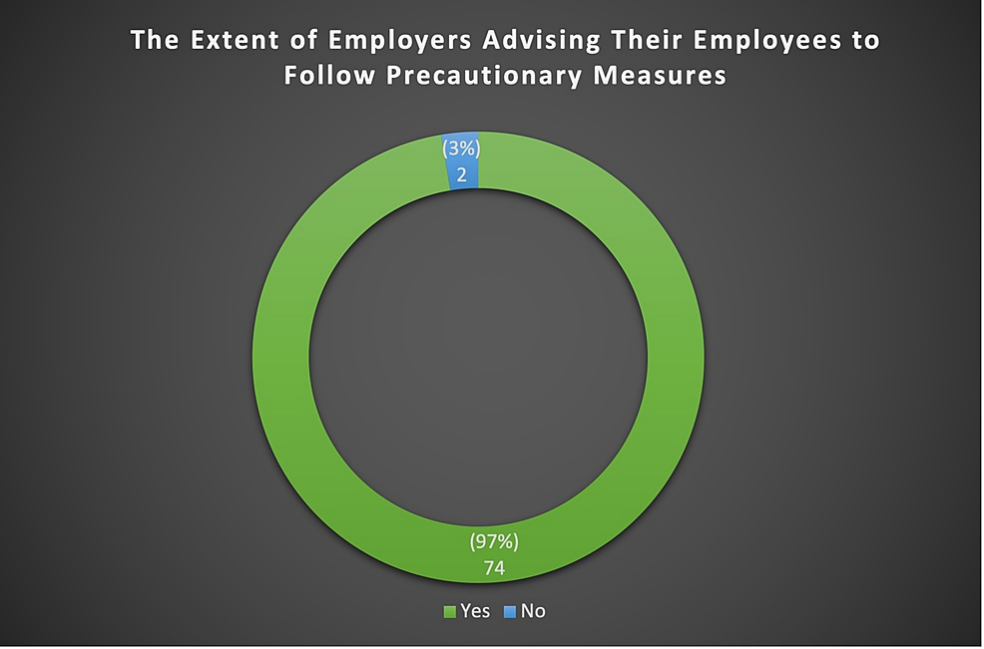
The extent of employers advising their employees to follow precautionary measures

Females were keener on taking care of their own health (83.4%), their family’s health, and the health of those around them (92.7%) compared to males, with a significant association (p-value = .028 and p-value = .002, respectively). More males than females reported that they could not commit to home quarantine due to the nature of their job (10.0% and 4.7%, respectively), and a statistically significant association was found (p-value = .002). About 94.9% of the total participants aged 59 years and above did not find any difficulty in committing to preventative measures. However, participants aged 15-25 years old indicated that they often forgot to adhere to preventative measures. As shown in Figure 1, 97% of the total assessed population of employers advised their employees to follow the precautionary measures while only 3% of them did not.

As presented in Table 3, 20.7% of males were engaged in healthcare-related volunteering platform/service delivery companies during the pandemic, and only 10.1% of females of the total population. The association was highly statically significant (p-value <0.001).

**Table 3:** Participants' registration in healthy volunteering platform/service delivery companies during the pandemic according to gender

Health Volunteering		Gender						Chi-square test
		Male		Female		Total		
		Count	Column N %	Count	Column N %	Count	Column N %	p-value
Have you registered on the healthy volunteering platform? Or any of the available activities such as service delivery companies and others?	Yes	66	20.7%	61	10.1%	127	13.8%	<0.001**
	NO	253	79.3%	540	89.9%	793	86.2%	
	Total	319	100.0%	601	100.0%	920	100.0%	
If yes, why? (Choose all that applied)								
I wanted to help sectors in need	Yes	39	59.1%	39	63.9%	78	61.4%	.575
	NO	27	40.9%	22	36.1%	49	38.6%	
	Total	66	100.0%	61	100.0%	127	100.0%	
I wanted to volunteer after my work/studies were suspended or stopped	Yes	16	24.2%	13	21.3%	29	22.8%	.694
	NO	50	75.8%	48	78.7%	98	77.2%	
	Total	66	100.0%	61	100.0%	127	100.0%	
I wanted to gain practice in my specialty and for my career	Yes	22	33.3%	15	24.6%	37	29.1%	.279
	NO	44	66.7%	46	75.4%	90	70.9%	
	Total	66	100.0%	61	100.0%	127	100.0%	
I wanted to go outside the house as I used to	Yes	9	13.6%	3	4.9%	12	9.4%	.093
	NO	57	86.4%	58	95.1%	115	90.6%	
	Total	66	100.0%	61	100.0%	127	100.0%	
To meet national needs and humanity appeal	Yes	55	83.3%	40	65.6%	95	74.8%	< .021>
	NO	11	16.7%	21	34.4%	32	25.2%	
	Total	66	100.0%	61	100.0%	127	100.0%	
Religious aspect	Yes	1	0.3%	1	0.2%	2	0.2%	.334b
	NO	65	98%	61	100%	126	99.8%	
	Total	66	100.0%	61	100.0%	127	100.0%	
If no, why? (Choose all that applied)								
I do not have time	Yes	70	27.7%	157	29.1%	227	28.6%	.683
	NO	183	72.3%	383	70.9%	566	71.4%	
	Total	253	100.0%	540	100.0%	793	100.0%	
Fear of getting the infection or transmitting it to my family	Yes	89	35.2%	199	36.9%	288	36.3%	.648
	NO	164	64.8%	341	63.1%	505	63.7%	
	Total	253	100.0%	540	100.0%	793	100.0%	
Because of my/family chronic illnesses	Yes	2	0.8%	5	0.9%	7	0.9%	.849b
	NO	251	99.2%	535	99.1%	786	99.1%	
	Total	253	100.0%	540	100.0%	793	100.0%	
I'm working in the health field already	Yes	5	2.0%	2	0.4%	7	0.9%	.024*b
	NO	248	98.0%	538	99.6%	786	99.1%	
	Total	253	100.0%	540	100.0%	793	100.0%	
Others	Yes	13	5%	45	8%	58	7%	-
	NO	240	95%	495	92%	735	93%	
	Total	253	100%	540	100%	793	100%	

## Discussion

The current research presents the Saudi community's perception of the precautionary measures during the COVID-19 pandemic in Saudi Arabia. Most respondents were pre-university and university-level females (Table 3).

The majority of participants expressed their commitment to continue washing their hands according to MOH instructions. The credit could be given to the Saudi MoH, and Saudi CDC attempts to promote adherence to precautionary measures through conducting several media awareness campaigns and guidelines [23]. However, similar findings were reported in that 72% of the African American population always wash their hands for at least 20 seconds and 21% wash their hands for at least 20 seconds for most of their time [24]. Moreover, this supports the observation that 84% of their study sample knew they had to wash their hands for 20 seconds according to MoH instructions [25].

When comparing gender with the participants' commitment to washing their hands for 40 seconds and sanitizing them for 20 seconds according to the Saudi MOH instructions, males were more likely to always be committed than females (66% to 58%) (p-value = 0.03). This is contrary to findings that showed that females were more likely to commit to precautionary measures during MERS-CoV [26].

Females were more likely to commit to using gloves when going outside the home compared to males (p-value <.001). In contrast, males were more likely to use face masks than females (p-value = .005). A significant difference between genders in face mask use wasn't reported in other studies [27].

When asked about practices at the workplace, male respondents reported washing their hands at work more than female respondents. This could be explained by the fact that female respondents were less likely to leave home during the pandemic and were more likely to work from home and do online shopping (p-value <.001). 

Postgraduate participants were more likely to always commit to washing their hands according to MOH instructions, use hand sanitizer, face masks, and practice social distancing than participants of lesser educational attainment. This is consistent with findings of another study by Baig, which found that higher educated participants were more likely to predict positive knowledge and attitudes towards the COVID-19 pandemic in Saudi Arabia [28].

Furthermore, respondents with pre-university education were found to be statistically significant for never leaving home during the pandemic's curfew. This could be largely attributed to homeschooling through virtual learning. Correspondingly, a study that examined students from a Polish secondary school indicated that females were less likely to leave home, use face masks, wear gloves, wash their hands, and avoid public places compared with males after the implementation of the legal regulations [29].

In this research, we inquired about sneezing etiquette as an important component of precautionary measures. The majority of the studied participants were always committed to the sneezing etiquette outlined by the Saudi MOH (74%), which could be largely attributed to the Saudi Centers for Disease Control and Prevention (CDC) and MOH for raising public awareness through social media platforms and websites about the novel coronavirus even before it reached Saudi Arabia [30-31]. In line with this, the study found that 75% of the participants knew that sneezing etiquette would prevent the COVID-19 virus from spreading [25].

Further, pre-university groups were more likely to answer “I don't know about sneezing etiquette” in comparison to higher educational groups. A similar finding was reported that most university students did not comply with the CDC sneezing standards [32]. With instant access to the latest global and local updates on the pandemic and the news, it seems that being educated could reinforce the implementation of precautionary measures.

Culturally, Saudi society observes strong family ties. This could have a double-edged impact on the perception of precautionary measures. From one end, precautionary measures could be adversely perceived and poorly implemented, as it was limiting the connectivity between members of the society. On the other hand, it could be utilized as an empowerment tool to further strengthen adherence to precautionary measures as people strive to protect members of their community. Participants were inquired about their commitment to advising people in their surroundings to follow preventive measures. Females were more likely to advise those who were not committed to nearby people compared to males.

Likewise, females were more likely to commit to home quarantine and apply preventive measures than males with a statistical association (p-value .028). This could be explained by the fact that females are rather wives and mothers with a strong commitment to look after their family members and an eager desire for the curfew to end so they can resume a normal life. Females were probably more vulnerable to pandemic fatigue due to feeling overwhelmed in adapting to the new norm with multiple responsibilities, including those related to online learning of their children and working from home. The study also mentioned that 32% of fathers who have children below 18 years old experienced or worsened their mental health due to fear of infection during the COVID-19 pandemic compared to 57% of mothers who can handle the disproportionately large burden of tasks [33].

Additionally, difficulties in the application and commitment to the preventive measures ranged from one age group to another. Younger participants, aged 15-25, were more likely to forget adhering to preventative measures (p-value .015) than other age groups. In contrast, participants aged 59 years and older expressed that faith in God is even more protective than adhering to precautionary measures (p-value .015).

Moreover, most participants indicated they would never commit, and only a few participants indicated they will always or usually commit to preventive instructions after lifting the total curfew. This could explain the rise of the precautionary measures violations that reached 31868 violations in March 2021 [34] and the increase of COVID-19 confirmed cases after roughly seven months of curfew lifting by the Saudi authorities [35]. As a result, the local officials needed to contain the second surge of COVID cases by reinforcing restrictions in public places and by limiting social gatherings for about 40 days until a fewer number of new cases was reported [36].

Remarkably, the university group was more likely to indicate they would never be committed after the end of the pandemic, which might be attributed to their excitement to return to their everyday life, including university/work and other entertaining activities, compared to the older age groups, for underestimating the likelihood of the future pandemic waves.

Strength 

The study gathered interesting findings of the Saudi community, which we were yet to know about. This would be the first study to address the perception of the Saudi community toward precautionary measures to combat COVID-19. Our intention was to understand the main force to drive the community, in general, to adhere to precautionary measures, hoping this will support the planning and implementation of future pandemic-related initiatives, which could be better customized to the local community to ensure full community engagement.

Limitations 

As this study was limited to the curfew period, we were required to distribute the questionnaire for a limited time during the lockdown period to ensure the acquisition of accurate responses. Despite offering the survey in the English language, we lacked responses from expat employers. Further, the sampling technique was limited to finding a significant difference in gender, educational levels, and employment categories, which are vital variables to implicate significant results.

Since data was collected through a self-administered questionnaire, recall bias and question misinterpretation may have occurred. The questionnaire was piloted for quality and clarity of questions prior to distribution. The small response rate was another limitation. A high response rate was difficult to achieve, but we enhanced our distribution daily by sending the questionnaire to different groups using different social media channels, including WhatsApp, Twitter, and Snap Chat, over two weeks.

## Conclusions

In conclusion, our research intended to measure the Saudi community’s perceptions toward preventive measures during the COVID-19 pandemic. The majority of our participants found it challenging to commit to precautionary measures outlined by the Saudi MoH, including handwashing, following a proper sneezing etiquette, and advising others to adhere to precautionary measures. Surprisingly, study respondents, most of whom are university graduates, indicated they would never commit to observing preventive instructions after the pandemic ended.

## Appendices

Questionnaire:

By agreeing to participate in this questionnaire, means that you are engaged in the study.All of your given information will be kept confidential for research purposes and will be destroyed after we finish the study. Your Participation Is Important To Have Public Opinion Towards COVID-19 Pandemic And People's Perception & Conviction Which Will Help Our Community To Raise Up So Please Express Your Opinions Honestly & Clearly.

S.1 Demographic Characteristics

Age

1= Below 15 years old

2= 15-25 years old

3= 26-36 years old

4= 37-47 years old

5= 48-58 years old

6= 59 years old and above

 Gender

1= Male

2= Female

Nationality

1= Saudi

2= Non-Saudi

Kingdom Region You Lived in

1= Central Region

2= Southern Region

3= Eastern Region

4= Northern Region

5= Western Region

Marital Status

1= Single

2= Married

3=Divorced/Separated

4= Widow

Monthly Income

1= Less than 5,000 Saudi Riyals

2= 5,000 - 10,000 Saudi Riyals

3= 11,000 - 15,000 Saudi Riyals

4= More than 15,000 Saudi Riyals

5= I don’t have a monthly income

Education

1= Illiterate

2= Pre-university Education

3= University Education

4= Post-graduate Education

Employment Sector

1= Governmental/Administrative

2= Governmental/Healthcare

3= Governmental/Education

4= Military

5= Private/Administrative

6= Private/Healthcare

7= Private/Education

8= Freelance/ Business Management (Employer)

9= Student

10= Others: ….

 If you are working in business administration, are you heading employees?

1= Yes

2= No

How many persons do you live with at home?

1 = I live alone

2= 1-3 persons

3 = 4-6 persons

4 = 7-9 persons

5 = 10 and more

6= Others: ….

Were you a citizen or resident who was returned from abroad?

1= Yes

2= No

Have you got infected by COVID19?

1= Yes

2= No

S2: Questions for Employer towards Applying Precautionary Measures

Determine the number of people you employ?

1 = 1-3

2 = 4-6

3 = 7-9

4 = 10 and more

5= Other: ….

Which type of housing do they live in?

1= Villa

2= Apartment

3= Shared house

4= Room

5= Other: ….

What housing qualification do they live in, terms of ventilation, construction, and occupational safety?

1= Qualified

2= Unqualified

3= Sort of

Have you trained your employees on occupational health/safety? (Such as machines, motors, and sharp tools)

1= Yes

2= No

3= Occupational safety only

4= Occupational health only

5= Not applicable

Did one of your employees got infected from COVID-19?

1= Yes

2= No

3= Don’t know

Have you advised your employees about the importance of precautions compliance?

1= Yes

2= No

If yes, why?

1= I care about them

2= I care about the community & the world’s safety

3= Fear of responsible actors questioning (accountability)

4= They’re my responsibility

5= Spreading my gained awareness

6= Other: ….

If not, why?

1= All of them are committed

2 = Media awareness is enough

3 = I was not obligated so I could advise them

4 = Other: ….

S3: COVID-19 Prevention Practice

Did you commit to wash your hands for 40 seconds and sanitize them for 20 seconds according to MOH instructions on “when to wash your hands?”

1= Yes Always

2= Usually

3= I commit to washing my hands but not according to instructions

4= I did not commit it at all

When you go to any place outside the home, what did you do?

1= I use gloves

2= I use a face mask

3= I use hand sanitizer when touching things

4= I wash my hands (soap) when touching things (At my workplace)

5= I Keep a safe distance from other persons

6= I don't do anything

7= Other: ….

Did you comply with the "sneezing etiquette" recommended by the Ministry of Health in cases of sneezing?

1= Always

2= Usually

3= Never

4= I don’t know it

When you receive or bring a thing from outside, what did you do? (Choose all that applied)

0= Not ticked

1.     I use gloves

2.     I use a face mask

3.     I Keep a safe distance from other people

4.     I sterilize my order

5.     I get rid of outer cases and cartoons

6.     I don't do anything

Have you advised whom around you to follow & commit to Precautionary measures?

1= Yes

2= No

If yes, why?

1= I care about them

2= To raise the awareness

3= People around me are not committed

4= I feel responsible towards my country and the world

5= Other: ……….

If not, why?

1= All of my nearby people are committed

2 = Media awareness is enough

3 = I was not committed myself to advise them

4 = Other: ……….

What would you do if you had early symptoms and wanted to make sure that you were infected with the virus?

1 = Searching through the internet for symptoms and causes

2 = Calling the unified number 937

3 = Use “Sehha” application for physician consultation

4= Use self-assessment service via “Mawid” application

5= Go to the nearest health facility

6= Waiting for more severe symptoms

7= I didn't do anything

What motivated you to commit to the home quarantine and applying Precautionary measures?

1 = Taking care of my health

2 = Taking care of the health of my family and those around me

3 = Taking care of the elderly and the chronically ill

4 = Commitment, patriotism, and community health

5 = I couldn't commit to (home quarantine) due to my work conditions

6 = I could not because I was influenced by those around me

7 = Not interested

8 = Not obligated

= Other: ….

What is the most motivating reason for you to be committed?

1= To avoid the increase of daily confirmed cases

2= Commitment to precautionary Measures

3= Desire for the curfew to end and resume normal life

4= In the spirit of “We Are All Responsible" slogan to protect the Kingdom

5= Taking care of the health of my family and those around me

6= Taking care of public health in general

7= Others: ….

If you find any difficulties applying and commit to precaution instructions, what was the reason?

1 = I didn't find any difficulty

2 = Being influenced by those around me

3 = I forget

4 = I feel bored

5= Unavailability of protective tools in my workplace

6 = Trust in Allah is enough

7= I think the virus is not that scary and I will be treated immediately 8= Other: ….

Will you commit with precautionary instructions even after the COVID-19 pandemic ends?

1= Always

2= Usually

3= Sometimes

4= Seldom

5= Never

If you choose (Always) or (Usually), why?

1.     I will be used to it

2.     I will be afraid that the virus will come back again

3.     society will be more cautious, so am I 

4.     I will maintain social distancing at work

5.     I will maintain social distancing on occasions

6.     I will not shake hands anymore

7.     I will keep a safe distance in public places

8.     I will avoid gatherings at work/malls/gyms

9.     I will lessen from gatherings at work/malls/gyms

10.  I will avoid family gatherings/occasions

11.  I will lessen from family gatherings/occasions

12.  I will eat at home

13.  Others: ….

If you choose (Sometimes) or (Seldom) or (Never), why?

1.     The virus will disappear when the crisis is over

2.     Faith in Allah is enough

3.     I will feel bored

4.     Others: ….

Have you registered on the healthy volunteering platform? Or any of the available activities such as delivery companies and others?

1= Yes

2= No

If yes, why?

1.     I wanted to help sectors in need

2.     I wanted to volunteer after my work/studies were suspended or stopped

3.     I wanted to gain practice in my specialty and for my career

4.     I wanted to go outside the house as I used to

5.     To meet national needs and humanity appeal

6.     Others: ….

If no, why?

1 = I do not have time

2 = Fear of getting the infection or transmitting it to my family

3 = Other: ….

S4:COVID-19   Pandemic Influence

Which of the following appointments have you postponed before or within the home quarantine?

1.     Hospital / primary care centers Periodic appointments

2.     Hospital / primary care centers Non- periodic appointments

3.     Surgical operations appointments

4.     Dental and cosmetic centers appointments

5.     Important travel dates

6.     External work appointments

7.     Internal work appointments

8.     I did not postpone any appointments

9.     I don’t have any appointments

10.  Others: …...

How does the e-learning/distance work decision affect your communication and interaction skills?

1.     It did not apply to me

2.     It saves time, effort, and money

3.     Difficulties in understanding and communicating

4.     Working all-day

5.     I don’t face any problems

6.     Other: ….

How does the decision to suspend prayers in the Holy Mosques and mosques affect your commitment to pray?

1 = Doesn’t affect me

2 = I delay my prayers after mosque prayers suspension

3 = I miss praying in the mosques

4 = I miss hearing Tarawih/Qiyam prayers

5 = I miss the Quran (Khatmah) in Masjid/Holy Mosques

6 = I miss performing Umrah

7 = It had a negative effect on my psychological wellbeing

8 = I have trouble getting zakat out of (gold and silver)

9 = I am not a Muslim

10 = Other: ….

How does this pandemic help you to manage your financial resources?

1.     Does not affect me

2.     Stop ordering from restaurants and cafes

3.     Stop/reduce going to malls and markets

4.     Stop/reduce the online ordering, such as food, clothes, and others

5.     Stop/reduce regular gatherings with family and friends

6.     I start to buy only necessary stuff after canceling the "cost of living allowance" decision

7.     I spend more money to buy food and cleaning supplies

8.     I spend more money than before

9.     I spend more money because of delivery fees

10.  Others: ….

How has this pandemic affected your physical exercises and healthy lifestyle?

1.     Doesn’t affect me

2.     I didn’t use to do physical exercises

3.     I didn’t use to follow a healthy lifestyle

4.     I had to cancel my subscription to healthy meals

5.     I had to cancel my gym subscription

6.     I have become more nervous and want to eat unhealthy food

7.     I find more time to focus on doing my routine physical exercises

8.     I have more desire to start doing physical exercise

9.     I find more time to start a healthy lifestyle

10.  I find more time to focus on my healthy lifestyle

11.  Others: ….

How has this pandemic affected your psychological health?

1.     Not affected

2.     I became anxious and stressed

3.     I wanted to be isolated from the community

4.     I stayed away from social media/press conferences for a while

5.     I got chronic diseases

6.     Sleeping disorders

7.     Other: ….

How did the home quarantine decision affect your relationships with those around you?

1.     It improved my relationship with my family

2.     It improved my relationship with my friends

3.     It improved my relationship with my study/work colleagues

4.     It weakened my relationship with my study/work colleagues/ friends

5.     It weakened my relationship with my family due to work pressure/overload

6.     It improved my relationship with my family and weakened it with my friends

7.     It improved my relationship with my friends and weakened it with my family

8.     Didn’t affect me at all

9.     Others: ….

Have you gained any new skills through the pandemic?

1= Yes

2 = No

If yes, please mention them?

What is your future prediction after this pandemic?

1 = Nothing

2 = Finance challenge (budget declining)

3= Occupational challenge (lack of jobs)

4= Changing of the educational system

5= Changing work style

6= Reoccurrence of the epidemic/other epidemics (God forbid)

7= Loss of control over the epidemic/other epidemics (God forbid)

8= Burden on the health system

9= Life will not return to what it used to be

10= Other: ….

What do you expect after the pandemic?

1.     Increase communication and social synergy

2.     Gaining new skills and habits such as healthy habits, savings, prevent extravagance, continue eating food from home, etc.

3.     More appreciation of things

4.     Other: ….

In Appreciation for Your Participation, Please Provide Us with Your Preference Way of Contacting Either (Mobile number, Or Email address) to Nominate You for A Prize Draw!

1= Name

2= City

3= Email Address

4= Mobile Phone

## References

[1] Fernandes N (2020). Economic effects of coronavirus outbreak (COVID-19) on the world economy. SSRN.

[2] Nicola M, Alsafi Z, Sohrabi C (2020). The socio-economic implications of the coronavirus pandemic (COVID-19): a review. Int J Surg.

[3] Fisher D, Wilder-Smith A (2020). The global community needs to swiftly ramp up the response to contain COVID-19. Lancet.

[4] Ebrahim SH, Ahmed QA, Gozzer E, Schlagenhauf P, Memish ZA (2020). Covid-19 and community mitigation strategies in a pandemic. BMJ.

[5] Lee VJ, Chiew CJ, Khong WX (2020). Interrupting transmission of COVID-19: lessons from containment efforts in Singapore. J Travel Med.

[6] Khalid T (2021). Saudi Arabia reports first coronavirus case, a Saudi national coming from Iran. Al Arabiya English. https://english.alarabiya.net/en/News/gulf/2020/03/02/Saudi-Arabia-reports-first-coronavirus-case-state-media.

[7] Yezli S, Khan A (2020). COVID-19 social distancing in the Kingdom of Saudi Arabia: bold measures in the face of political, economic, social and religious challenges. Travel Med Infect Dis.

[8] (2021). Saudi Arabia’s measures to combat coronavirus since February: a timeline. Al Arabiya English. Al Arabiya English.

[9] Khalil R, Mansour AE, Fadda WA (2020). The sudden transition to synchronized online learning during the COVID-19 pandemic in Saudi Arabia: a qualitative study exploring medical students' perspectives. BMC Med Educ.

[10] Arora G, Kroumpouzos G, Kassir M (2020). Solidarity and transparency against the COVID‐19 pandemic. Dermatol Ther.

[11] Ivic S (2020). Vietnam's response to the COVID-19 outbreak. Asian Bioeth Rev.

[12] Ali MA, Al-Khani AM, Sidahmed LA (2020). Migrant health in Saudi Arabia during the COVID-19 pandemic. East Mediterr Health J.

[13] Addawood A, Alsuwailem A, Alohali A (2020). Tracking and understanding public reaction during COVID- 19: Saudi Arabia as a use case. OpenReview.

[14] (2021). Saudi Gazette. MoH designates March 2 as ‘Health Martyr Day’. Saudi Gazette.

[15] (2021). Saudi Arabia to provide free COVID-19 vaccines in pharmacies: health minister. Al Arabiya English. https://english.alarabiya.net/coronavirus/2021/03/03/Coronavirus-Saudi-Arabia-to-provide-free-COVID-19-vaccines-in-pharmacies-Health-minister.

[16] (2021). General Authority for Stats. KSA. https://www.stats.gov.sa/en/indicators/1.

[17] (2021). OpenEpi. Sample size for frequency in a population. https://www.openepi.com/SampleSize/SSPropor.htm.

[18] Brown G. Natalie Low (NAO), Ramona Franklyn (DfE), Emma Drever (DfT) Catherine Mottram (DfT (2021). GSR quota sampling guidance. https://gss.civilservice.gov.uk/wp-content/uploads/2018/03/Quota-sampling-guidance-4.pdf.

[19] (2021). Wheel of Names. https://wheelofnames.com.

[20] (2021). SurveyMonkey. https://www.surveymonkey.com.

[21] (2021). Microsoft Corporation. Microsoft Excel 2016. Microsoft Excel.

[22] (2021). IBM. Statistical Package for Social Sciences (PASS statistics). International Business Machines Corporation (IBM). https://www.ibm.com/products/spss-statistics?lnk=ushpv18ct7.

[23] (2021). MoH launches COVID-19 awareness website. Saudi Gazette. https://saudigazette.com.sa/article/593355.

[24] Block R Jr, Berg A, Lennon RP, Miller EL, Nunez-Smith M (2020). African American adherence to COVID-19 public health recommendations. Health Lit Res Pract.

[25] Siddiqui AA, Alshammary F, Amin J, Rathore HA, Hassan I, Ilyas M, Alam MK (2020). Knowledge and practice regarding prevention of COVID-19 among the Saudi Arabian population. Work.

[26] ALdowyan N, Abdallah AS, El-Gharabawy R (2017). Knowledge, attitude, and practice (KAP) study about middle east respiratory syndrome coronavirus (MERS-CoV) among the population in Saudi Arabia. Int Arch Med.

[27] Howard MC (2020). Gender, face mask perceptions, and face mask wearing: are men being dangerous during the COVID-19 pandemic?. Pers Individ Dif.

[28] Baig M, Jameel T, Alzahrani SH (2020). Predictors of misconceptions, knowledge, attitudes, and practices of COVID-19 pandemic among a sample of Saudi population. PLoS One.

[29] Guzek D, Skolmowska D, Głąbska D (2020). Analysis of gender-dependent personal protective behaviors in a national sample: Polish Adolescents' COVID-19 Experience (PLACE-19) study. Int J Environ Res Public Health.

[30] (2021). Public Health Authority. Community & public. https://covid19.cdc.gov.sa/community-public/.

[31] (2021). MoH. COVID-19 guidelines. https://www.moh.gov.sa/en/Ministry/MediaCenter/Publications/Pages/covid19.aspx.

[32] Berry TD, Fournier AK (2014). Examining university students' sneezing and coughing etiquette. Am J Infect Control.

[33] Power K (2020). The COVID-19 pandemic has increased the care burden of women and families. Sustain Sci Pract Policy.

[34] (2021). Numbers and statistics. Unified National Platform COVID-19. https://covid19.my.gov.sa/ar/Sectors/security/Pages/default.aspx.

[35] SPA SPA (2021). Public / health spokesperson: we are monitoring an increase in the confirmed cases and we call everyone to raise vigilance and adhere to the precautionary measures. Saudi Press Agency. https://www.spa.gov.sa/2183273.

[36] SPA SPA (2021). Public / responsible source at The Ministry of Interior. It was decided not to extend the precautionary measures from next Sunday, with an exception of some measures. Saudi Press Agency. https://www.spa.gov.sa/viewfullstory.php?lang=ar&newsid=2197400.

